# Supplementary material for: The interactome of intact mitochondria by cross-linking mass spectrometry provides evidence for coexisting respiratory supercomplexes
Source: Mol Cell Proteomics. 2017 Dec 8;17(2):216–32. doi: 10.1074/mcp.RA117.000470 (PMC5795388; doi:10.1074/mcp.RA117.000470)
Supplement: Supplemental Data [file supp_RA117.000470_133922_0_supp_23978_fzffwf.zip › spectra_annotation/mito_spectra_annotation/139-1-2-1-4-1.pdf]

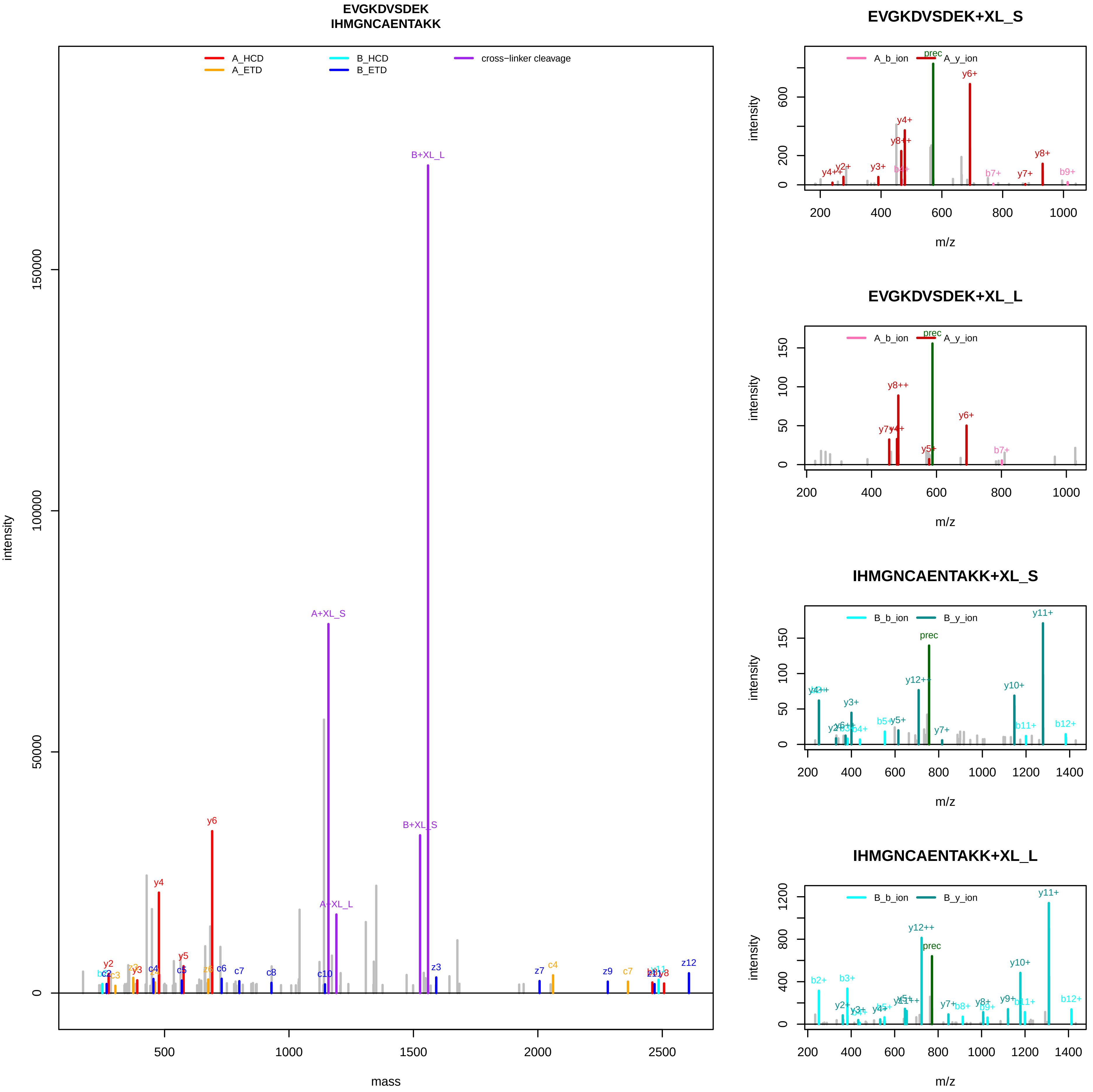
EVGKDVSDEK
IHMGNCAENTAKK
A_HCD
A_ETD
B_HCD
B_ETD
cross−linker cleavage
B+XL_L
A+XL_S
B+XL_S
A+XL_L
y6
y4
y5
y2
y3
c2
c3
c4
c5
c6
c7
c8
c10
z3
z7
z9
z12
intensity
mass
0
50000
100000
150000
500
1000
1500
2000
2500
EVGKDVSDEK+XL_S
A_b_ion
A_y_ion
prec
y6+
y4+
y8++
y3+
y2+
y4++
y7+
y8+
b7+
b9+
intensity
m/z
0
200
600
200
400
600
800
1000
EVGKDVSDEK+XL_L
A_b_ion
A_y_ion
prec
y8++
y6+
y5+
b7+
intensity
m/z
0
50
100
150
200
400
600
800
1000
IHMGNCAENTAKK+XL_S
B_b_ion
B_y_ion
y11+
prec
y12++
y10+
y3+
y5+
y7+
b12+
intensity
m/z
0
50
100
150
200
400
600
800
1000
1200
1400
IHMGNCAENTAKK+XL_L
B_b_ion
B_y_ion
y11+
y12++
prec
y10+
b2+
b3+
y2+
y4+
y7+
y8+
y9+
b12+
intensity
m/z
0
400
800
1200
200
400
600
800
1000
1200
1400
